# Molecular Dynamics Simulations Demonstrate Reduced Antibiotic Affinity to Mirror Bacterial Targets

**DOI:** 10.34133/csbj.0198

**Published:** 2026-09-03

**Authors:** Paul-Enguerrand Fady, Juliette Ciccone

**Affiliations:** ^1^ The Centre for Long-Term Resilience, London WC2H 9JQ, UK.; ^2^ University College London, London WC1H 0AJ, UK.

## Abstract

“Mirror life”, self-replicating organisms composed of nonnatural-chirality biomacromolecules, presents a future threat with potentially global consequences. Consequently, there is strong agreement among experts that it should not be created. However, there is some disagreement over how effective existing medical countermeasures might prove against mirror bacteria, in the event that they were created. Here, we leverage computational chemistry methods including docking and molecular dynamics to determine the likely binding efficacy of existing antibiotics against natural and mirror bacterial protein targets. We find that most existing antibiotics fail to bind to mirror bacterial protein targets, unlike their natural-chirality targets. This suggests altered binding of current medical countermeasures, which may impact antimicrobial activity against mirror bacteria if the latter were created.

## Introduction

Living organisms are composed of biomacromolecules with a specific chirality. All known life-forms exhibit homochirality: glycans are predominantly composed of sugars in the D-configuration (such as D-glucose, D-mannose, and D-galactose), i.e., sugars with right-handed chiral centers [1]. The D-2-deoxyribose of DNA is also chiral, imparting a right-handed directionality to the helical twist [2]. Conversely, proteins in living organisms are composed predominantly of L-amino acids, i.e., ones with left-handed chiral centers [3]. While it is possible for monomers of opposite chirality to be integrated into biomacromolecules (and even common in, e.g., peptidoglycan bacterial cell walls, which contain D-alanine and D-glutamate) [4], the predominant pattern of chirality is fixed in one specific form for individual biomacromolecules.

“Mirror life”, or synthetic living organisms composed of biomacromolecules built using the nonnaturally occurring enantiomers of biomolecular monomers, presents an alternative option to this universal truth. Recent advances in synthetic cell biology have transformed mirror life from a theoretical science fiction scenario to a material future threat. Synthesizing life from matter remains an unsolved scientific problem, which, if solved, would enable the creation of natural-chirality life as well as mirror life. While the exact timeline for the development of self-replicating mirror organisms is disputed, experts estimate that it could be achieved within the next 10 to 30 years, depending on resource allocation [5].

Mirror life could represent a future threat with potentially global consequences. A technical report published in December 2024 outlines the ways in which mirror bacteria could subvert human, animal, and plant immune systems, leading to uncontrolled growth due to lack of recognition by stereospecific defenses [5]. Were mirror bacteria to be created and find their way into the environment—whether through deliberate release or a containment breach—they could proliferate extensively. This could ultimately lead to nutrition depletion in key ecological niches, starving keystone species of nutrients, which could drive severe downstream effects.

The environmental picture motivates policy recommendations to ban research that directly leads to the creation of mirror life. This is distinct from research that may involve mirror macromolecules, which must be permitted in order to foster innovation in life and materials sciences. There is widespread agreement among scientific experts from a range of domains that mirror life should not be created, as noted in the Policy Forum piece accompanying the 2024 technical report [5], the “Spirit of Asilomar” conference statement on mirror life in early 2025 [6], the *Paris Conference on Risks from Mirror Life Meeting Report* from mid-2025 [7], a commentary by the German Central Commission for Biological Safety [8], and a report from the United Nations Educational, Scientific and Cultural Organization International Bioethics Committee [9].

However, there has been pushback from some experts surrounding the actual level of risk associated with self-replicating mirror organisms, such as mirror bacteria. A small minority of dissenting voices have indicated their belief that current medical countermeasures such as antibiotics would successfully neutralize the threat of mirror organisms by virtue of their nonstereospecific activity.

There is early evidence that antibiotics would not be effective against mirror bacteria. In the absence of mirror bacteria against which to directly test compounds, 2 parallel strategies have emerged to determine the likely efficacy of current countermeasures. The first is to synthesize mirror-chirality antibiotics and test them against native bacteria. This strategy relies on the assumption of equivalency between the interaction of mirror antibiotics with native-chirality bacteria and native-chirality antibiotics with mirror bacteria [10]. While this provides useful experimental evidence, it leverages costly enantiomers of antibiotics and requires “wet-lab” resources, which constrain the throughput of experiments.

The other strategy is to investigate antibiotic efficacy against mirror bacteria utilizing computational chemistry. Thus far, docking and molecular dynamics have been used to investigate the efficacy of amoxicillin (AMX) against native-chirality and mirror bacterial protein targets from *Chlamydia trachomatis* and *Staphylococcus aureus* [11,12]. Computational approaches enable investigations into this sensitive topic in a risk-free manner, i.e., without generating any mirror components that may contribute to the development of mirror life. In addition, this strategy provides a reproducible and iterative workflow that can be cheaply and rapidly adapted to study a range of targets with minimal resource requirements. This provides a risk-free environment for high-throughput determination of binding efficacy. A reduced binding efficacy may plausibly have a negative effect on antimicrobial activity—although this work is strictly computational and does not present *in vitro* validation of results.

In this work, we have chosen to leverage exclusively the computational chemistry approach described above. While experimental validation of the results herein is of course desirable, they are outside the stated scope of our work, which is strictly computational. By applying molecular dynamics and docking to help elucidate interactions between native and mirror proteins with antibiotic ligands, we have built on the results of previous computational studies. Other groups have studied the interaction of penicillin-binding protein 3 (PBP3) from *C. trachomatis* with AMX [11]. Our first aim, therefore, was to reproduce the results of these experiments with the exact same simulation setup: ligand, protein model, force field, etc. This provided a baseline of confidence in our study methodology and in the findings of previous work.

**Fig. 1.**
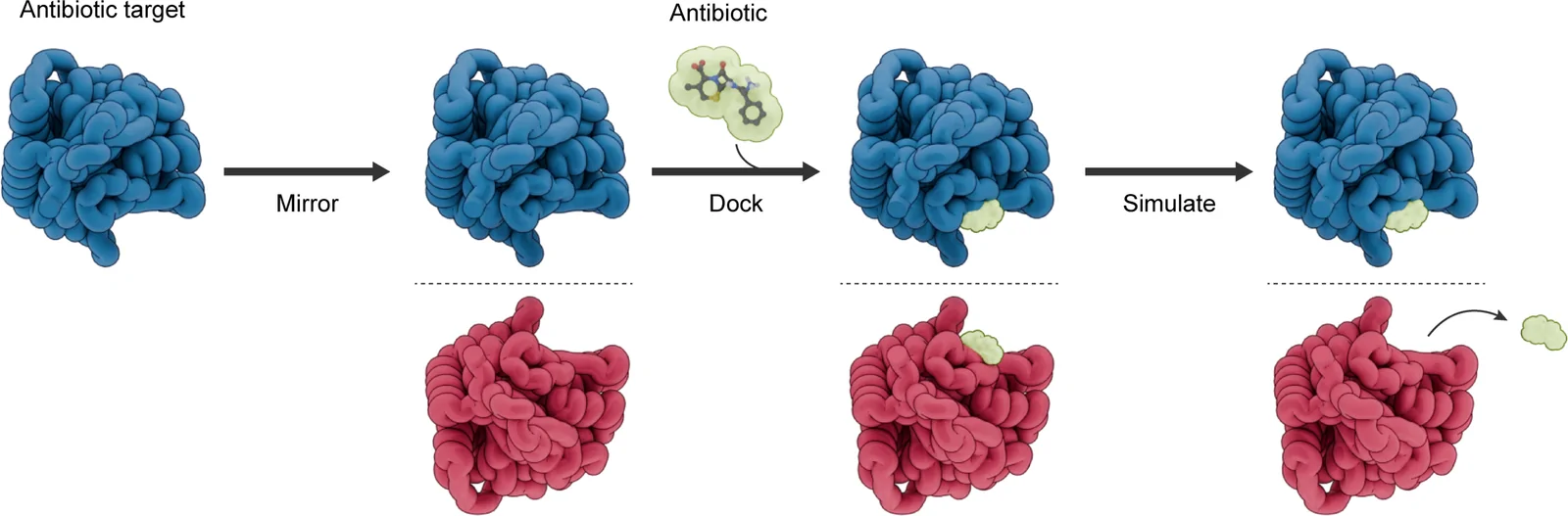
Computational chemistry enables detailed investigations of interactions of antibiotics and their chiral targets, native and mirrored. Computationally rendered schematic diagram depicting the experimental workflow: antibiotic target proteins from pathogenic species were computationally mirrored, to emulate proteins assembled from D-amino acids in mirror life-forms. The native, i.e., L-amino acid (navy), and mirror, i.e., D-amino acid (scarlet), proteins were simulated at equilibrium, before performing molecular docking with their constituent antibiotics. Further unrestrained molecular dynamics were performed to determine if the antibiotic would remain in its binding site, suggesting that it could inactivate the target protein, and function as an antimicrobial.

Doing so allowed us to meet our second aim: to determine whether the conclusions of previously published academic studies were generalizable across antibiotics with differing physicochemical characteristics. We examined this question by extending a well-established computational methodology to a novel range of antibiotics and bacterial protein targets (Fig. 1). This provides novel, robust *in silico* data that motivate further research into the efficacy of existing countermeasures against hypothetical mirror organisms by the field at large—including through future experimental work that may mirror existing laboratory-based approaches [10].

## Materials and Methods

### Experimental procedure

Pre-equilibrated antibiotic protein targets and their respective mirror images were simulated in the presence of their antibiotic ligand, using continued ligand proximity as a proxy for target inhibition. High-resolution structural data for antibiotic targets were gathered from the Protein Data Bank (PDB) and computationally mirrored. Both mirror and native proteins were then computationally equilibrated, after which their constituent antibiotic was inserted into the binding site via molecular docking. Subsequently, proteins were further simulated in the presence of the antibiotic ligand to compare the potential relative affinity of the antibiotics for the native and mirror form targets.

### Simulation preparation

**Fig. 2.**
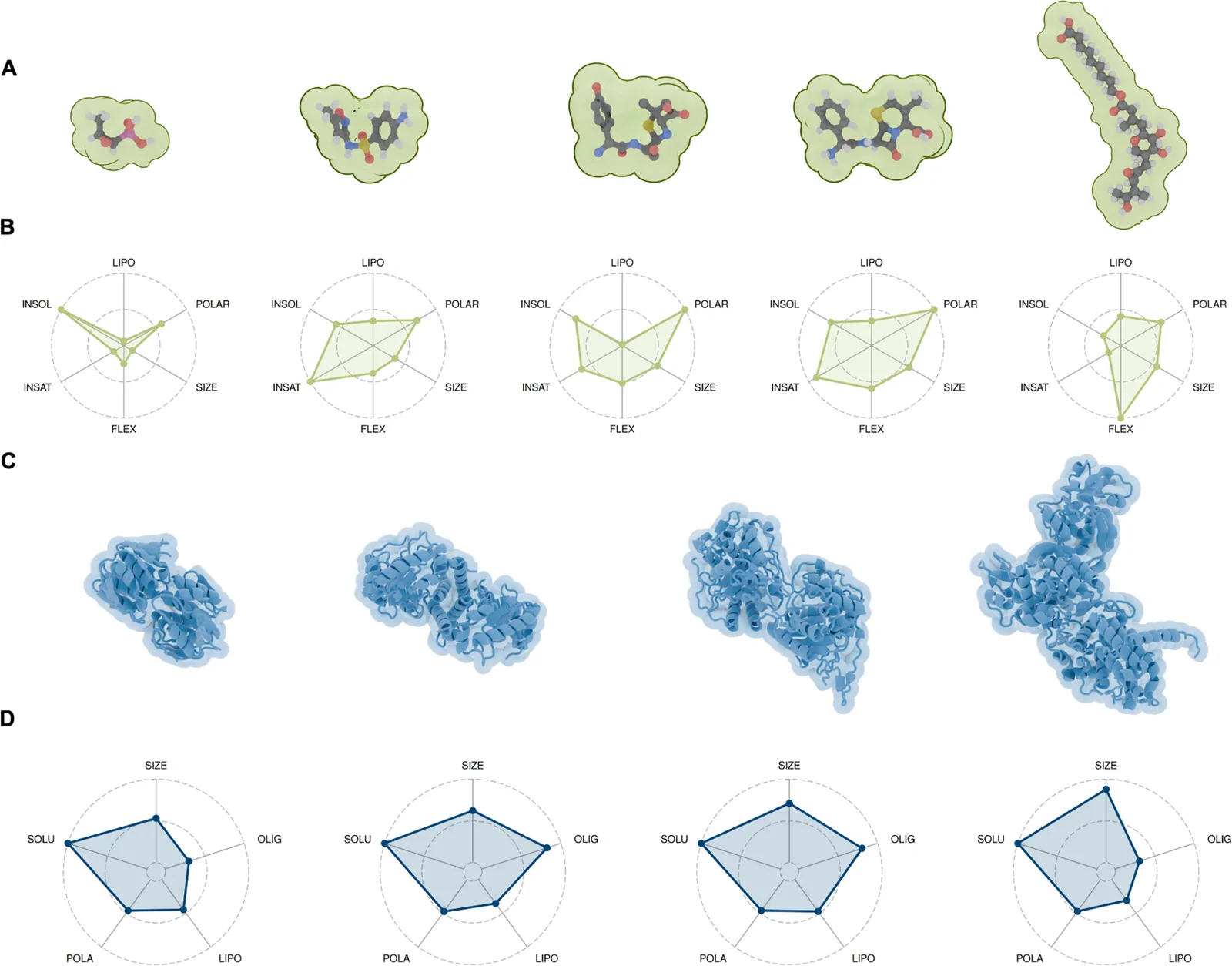
Antibiotics and targets of differing physicochemical and structural properties that were investigated in this work. (A) 3-dimensional (3D) molecular conformers (carbon atoms are depicted as gray; hydrogen, white; oxygen, red; sulfur, yellow; and nitrogen, blue; electrostatic shells are indicated in green) and (B) physicochemical properties of antibiotics used in this study, as assessed by SwissADME [81]. The parameters are lipophilicity (octanol–water partition coefficient [XLogP]) [82], polarity (topological polar surface area [TPSA]) [83], size (molecular weight), insolubility (Delaney estimated solubility [ESOL]) [84], insaturation (fraction Csp3), and flexibility (number of rotatable bonds). Left to right shows fosfomycin (FFQ; CID: 446987), sulfamethoxazole (SMX; CID: 5329), amoxicillin (AMX; CID: 33613), cephalexin (CPX; CID: 27447), and mupirocin (MRC; CID: 446596). (C) Space-filling and cartoon structures for the 4 proteins investigated in this study. Left to right is UDP-*N*-acetylglucosamine 1-carboxyvinyltransferase from *Enterobacter cloacae* (MurA; PDB: 3lth) [14], dihydropteroate synthase from *Staphylococcus aureus* (DHPS; PDB: 1ad1) [15], the transpeptidase domain of penicillin-binding protein 3 from *S. aureus* (PBP3; PDB: 6i1f) [16], and isoleucyl-tRNA synthetase from *Thermus thermophilus* (IleRS; PDB: 1jzs) [17]. (D) Structural properties of the antibiotic protein targets studied. The molecular weight (SIZE) in kilodaltons, normalized between 10 and 240 kDa [85]. The number of protein chains (OLIG). The exterior lipophilicity (LIPO), normalized between 0 and 1 [86], calculated using the Wimley–White method [87]. The average solvent accessible residue polarity (POLA), scaled to between 0 and 80 [88]. The protein solubility (SOLU) calculated as the inverse Boman index [89], normalized between 0 and 4.

The protein targets of antibiotics and their mirror images were first simulated at equilibrium. All-atom protein files for a selection of antibiotic targets were downloaded from the PDB [13]. The proteins investigated were UDP-*N*-acetylglucosamine 1-carboxyvinyltransferase from *Enterobacter cloacae* (MurA; PDB: 3lth) [14], dihydropteroate synthase from *S. aureus* (DHPS; PDB: 1ad1) [15], the transpeptidase domain of PBP3 from *S. aureus* (PDB: 6i1f) [16], and isoleucyl-tRNA synthetase from *Thermus thermophilus* (IleRS; PDB: 1jzs) [17] (Fig. 2). Mirror image protein structures were generated using a Python protocol developed by Pedroni *et al.* [11,12]. The script applies a full spatial inversion through the origin for every ATOM and HETATM coordinate in a given PDB file, e.g., (*x*, *y*, *z*) becomes (−*x*, −*y*, −*z*), producing a mirror image of the structure. As a result, all stereocenters are inverted and the “handedness” of the molecule is reversed. This converts the naturally occurring L-amino acid configurations (typically S for residues except cysteine) to the corresponding D-amino acid configuration [18]. This transformation is applied to only the molecular coordinates and not the topology, so atomic connectivity remains unchanged. Inversion of the desired coordinates was verified via inspection of the outputted .pdb files, as well as subsequent visualization, see Fig. S6 for a demonstration of the stereochemical inversion. Structures were then solvated in SPC/E water and neutralized with Na^+^ and Cl^−^ ions using GROMACS tools (version 2024.3) [19]. Structures were minimized to avoid steric clashes using a steepest descent algorithm for a maximum of 10,000 steps. Systems were then equilibrated to 300 K for 100 ps, using a velocity-rescaling thermostat with a 2-ps coupling time. This was followed by isobaric equilibration to 1 bar using isotropic Parrinello–Rahman pressure coupling for 100 ps, also with a 2-ps coupling time (Fig. S4). Production simulations were performed for at least 100 ns with at least 2 repeats. The CHARMM36 force field [20] was used and electrostatic forces were calculated using the particle-mesh Ewald algorithm, and a smooth switching algorithm with a 1.2-nm cutoff was used for van der Waals and electrostatic interactions.

### Molecular docking

Equilibrated proteins were then docked with their respective antibiotics. Equilibration of native and mirror proteins was assessed by stabilization of the c-alpha root mean squared deviation (RMSD) and radius of gyration (RoG). Once equilibrated, representative protein structures were prepared for docking and then docked with antibiotic ligands. Protein structures had been relieved of any crystallographic components, e.g., waters or cocrystallized ligands prior to equilibration. To prepare for docking, any nonprotein components of the equilibration simulations, such as water or ion atoms, were removed. Ionizable amino acid side chains were maintained from the equilibration, matching physiological conditions at pH 7.5. Protein receptors were then converted to PDBQT format using Meeko [21], with polar hydrogens and Gasteiger partial charges added. Amino acid side chains were treated as rigid during docking, as the proteins were already equilibrated, and the ligand-binding pockets were previously identified. Three-dimensional molecular conformations for the ligands were downloaded from PubChem [22]. The ligands used were sulfamethoxazole (SMX; CID: 5329), fosfomycin (FFQ; CID: 446987), AMX (CID: 33613), cephalexin (CPX; CID: 27447), and mupirocin (MRC; CID: 446596). Ligand tautomers were also protonated at pH 7.5 and converted to PDBQT format with Gasteiger charges ready for docking using Meeko [21]. Rotatable bonds in the ligands were retained as flexible during the docking. Mirror ligands and mirror proteins were treated identically to their native counterparts to ensure equivalent treatment of stereochemistry, protonation, and charge assessment during docking calculations. Antibiotic-binding sites were identified using the bound antibiotics from the crystallographic data. In the case of DHPS, where there was no bound ligand, a homolog with the correctly bound ligand was used (PDB: 3tzf) [23] and structures were aligned using TM-align, where necessary [24]. The docking grid box was centered on the crystallographic ligand centroid. Grid dimensions were generated automatically using the Meeko receptor preparation utility with a 1.5-Å padding in each dimension around the reference ligand coordinates. Docking was then performed using AutoDock Vina [25], using a random seed; exhaustiveness was set to 8; the energy range was set at 3 kcal/mol; and the number of modes inspected was 10 [26]. Prior to docking experiments, redocking of the crystallographic ligands against the antibiotic target proteins was performed to validate the docking procedure, revealing an average heavy atom RMSD of 0.22 ± 0.018 nm.

### Ligand simulations

Docking poses with the lowest energy minima were taken forward for ligand-bound simulations. Ligand parameters were generated using CGenFF [27] implemented in CHARMM-GUI. All parameters had penalty scores <10, suggesting high analogy to existing parameters, and further optimization was not required. System topologies were generated using Gromologist [28], before being prepared, equilibrated, and simulated as described above. Ligand-bound trajectories were again simulated for at least 100 ns with a minimum of 2 repeats.

### Analysis

Analysis was performed using GROMACS tools. To reduce the impact of equilibration effects, the initial 70% of the trajectories were discarded when performing time-averaged analysis such as RMSD or RoG analysis. PLIP was used to identify key binding residues [29], LigPlot+ was used to generate 2-dimensional residue interaction maps [30], and renders were produced using VMD (version 1.9.4a55) [31] and Blender (version 5.0), including the Molecular Nodes add-on. Data analysis and plotting was performed using R and ggplot2 [32,33], including the packages peptides (version 2.4.6), ggradar (version 0.2), bio3d (version 2.4-5), and fmsb (version 0.7.6) [34–37].

## Results

### Enolpyruvyl transferase

Enolpyruvyl transferase (MurA) is the smallest enzyme tested; it acts to catalyze the first step in bacterial cell wall biosynthesis [38]. MurA is a UDP-*N*-acetylglucosamine enolpyruvyl transferase, which generates UDP-GlcNAc-enolpyruvate and inorganic phosphate from phosphoenolpyruvate (PEP) and UDP-*N*-acetylglucosamine [39]. The MurA investigated in this work is a 45.58-kDa monomeric protein of 2 globular domains from the gram-negative bacteria *E. cloacae*. This species is one of the most common *Enterobacteriaceae*, and acts as an opportunistic pathogen [40]. *Enterobacteriaceae* infections can result in bacteremia, sepsis, meningitis, endocarditis, and osteomyelitis as well as infections in the urinary and respiratory tracts [41–43].

When simulated in solution, both the native and mirror forms of MurA rapidly equilibrated. Equilibration status was determined by assessing the RMSD and RoG values of alpha carbons in the protein. Both the native and mirror forms appeared geometrically stable after the 100-ns equilibration, consistent with previous reports of simulating proteins this size from crystallography data [44,45]. After equilibration, native MurA reported an RMSD value of 0.16 ± 0.02, while the mirror form reported an increased 0.35 ± 0.1 nm. Equilibration of the structure was further confirmed by the RoG analysis, which rapidly stabilized to 2.23 ± 0.004 and 2.31 ± 0.02 nm (Fig. S1).

MurA is inhibited by the action of the antibiotic FFQ. FFQ is a 1.38-kDa broad-spectrum antibiotic. The antibiotic acts as a competitive inhibitor, entering the PEP binding site and alkylating Cys115 [46]. Cys115 is located within a highly mobile loop within the enzyme’s active site [47] and is an essential component in the catalytic action of MurA [48] and conserved in all bacterial homologs [49], rendering it an excellent antibiotic target. Alkylation of Cys115 also forces the enzyme into an inactive, closed conformation, irreversibly blocking peptidoglycan synthesis, which is essential for bacterial survival [50].

**Fig. 3.**
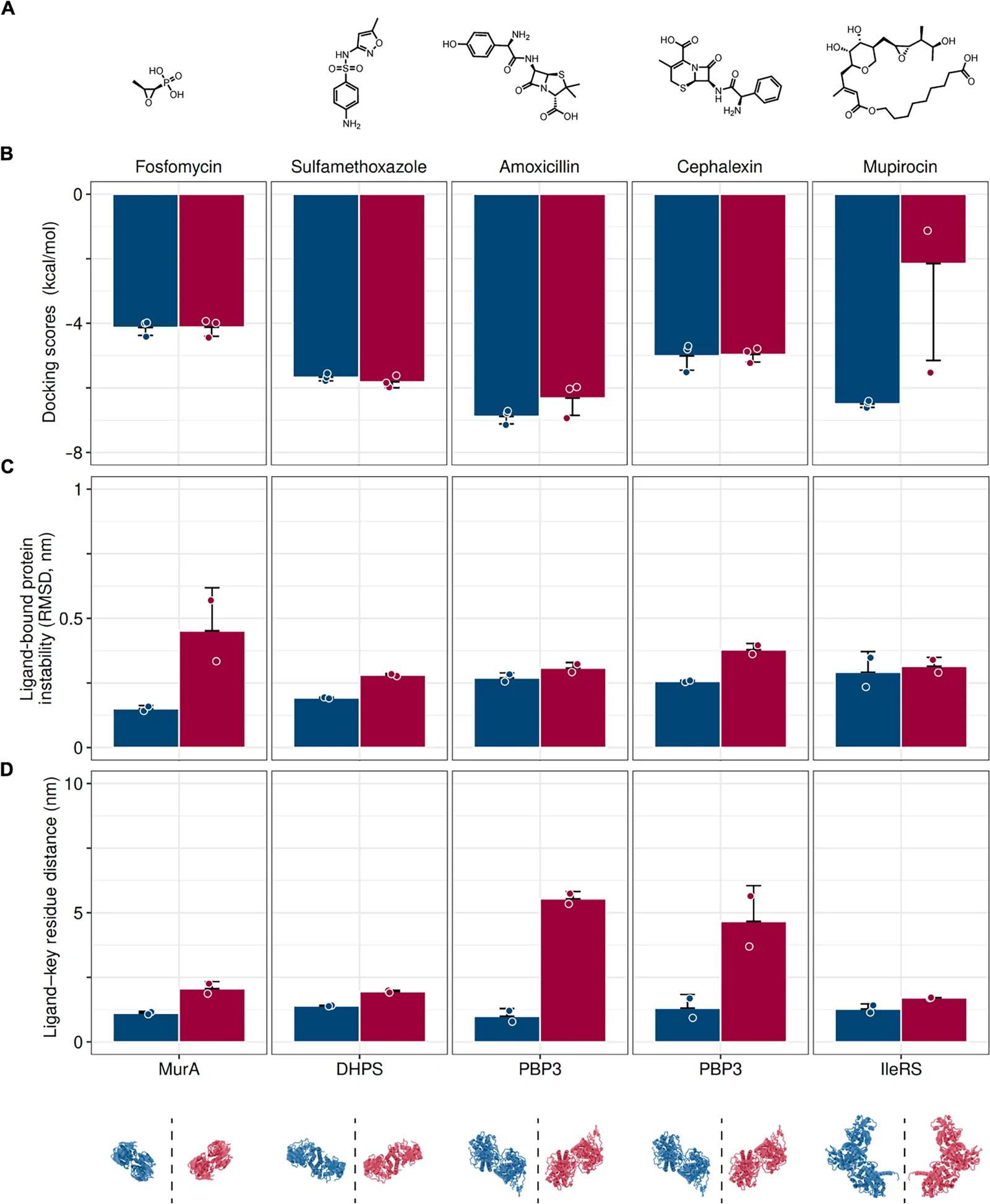
Antibiotics are not effective against mirror targets when compared to their native states, even when well fitted inside the target binding pocket. (A) Chemical structures for the antibiotics investigated. (B) Docking scores derived when docking antibiotics into the equilibrated native (navy) and mirror (scarlet) targets. (C) Protein stability, as measured by alpha carbon root mean squared deviation (RMSD), when simulated after docking with the bound relevant antibiotic. (D) Distance between the antibiotic ligand and the key residue involved in antibiotic inactivation. Each graph shows the average of at least 2 repeats; the error bars show the standard deviation, and the points show the individual data. The data shown here were time-averaged over the terminal 30% of each trajectory. The key residues, determined using PLIP and LigPlot+, as well as literature data, are as follows: enolpyruvyl transferase (MurA)–fosfomycin, Cys115; dihydropteroate synthase (DHPS)–sulfamethoxazole, Thr51; penicillin-binding protein 3 (PBP3)–amoxicillin, Ser320; PBP3–cephalexin, Ser320; and isoleucyl-tRNA synthetase (IleRS)–mupirocin, Leu583.

Docking of FFQ into the active site of MurA was remarkably similar in the native and mirror forms. In both cases, the antibiotic fit into a similar groove in the active site, likely due to the flexibility of the Cys115-containing loops, demonstrated by comparable docking scores of −4.13 ± 0.24 and −4.12 ± 0.28 kcal/mol (Fig. 3B) for the native and mirror forms, respectively, which are in-line with previous hybrid quantum mechanical/molecular mechanical (QM/MM) calculations of FFQ-modifying enzymes [51].

**Fig. 4.**
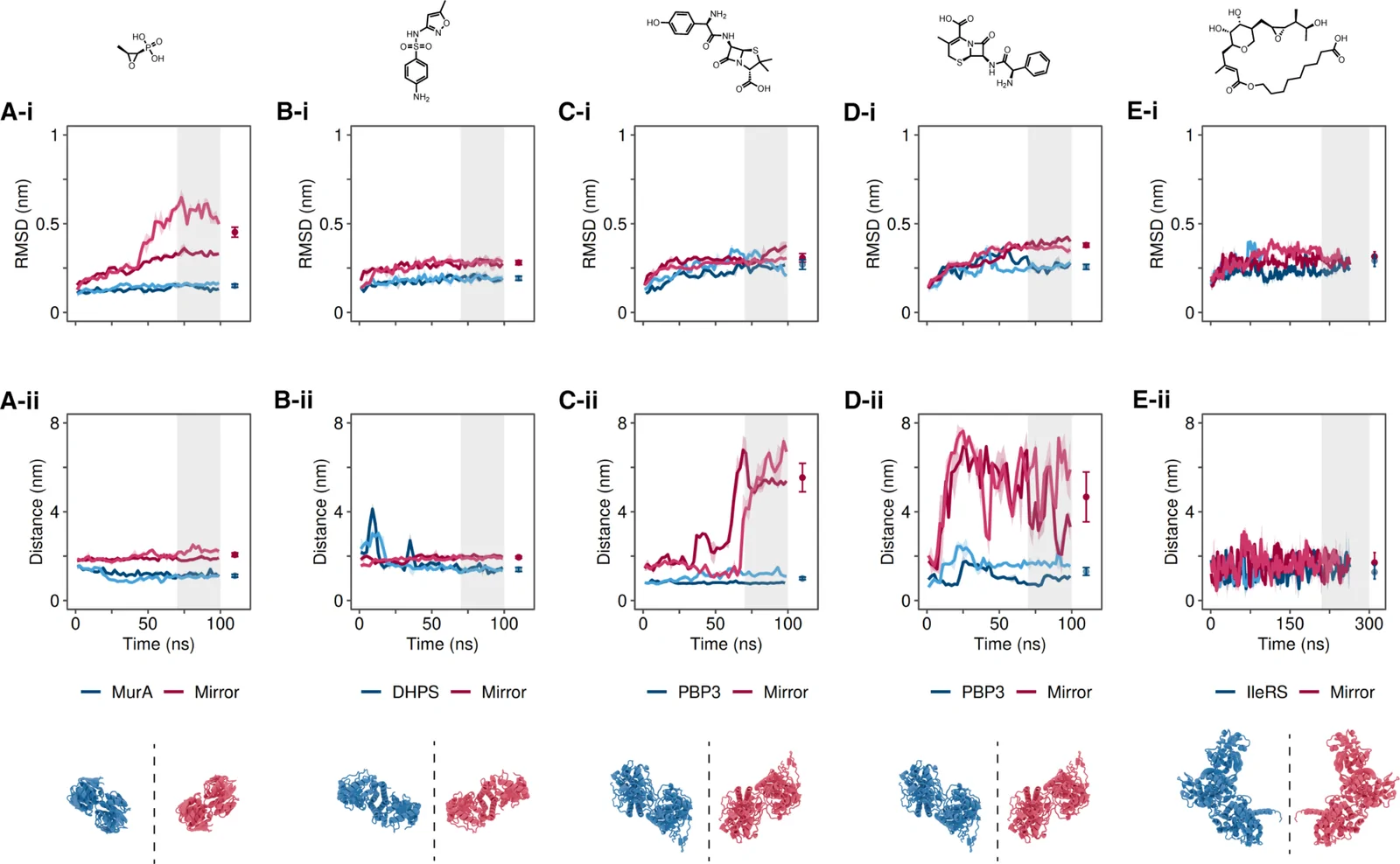
Stability and key residue interactions of antibiotics with their targets and mirrors are antibiotic and target dependent. (A) Chemical structure of fosfomycin: (i) time-averaged alpha carbon root mean squared deviation (RMSD) for enolpyruvyl transferase (MurA; navy) and the mirror form (scarlet) once docked with fosfomycin; (ii) distance between fosfomycin and its key target residue. (B) (i and ii) As above, for dihydropteroate synthase (DHPS) and sulfamethoxazole. (C) (i and ii) Penicillin-binding protein 3 (PBP3) and amoxicillin. (D) (i and ii) PBP3 and cephalexin. (E) (i and ii) Isoleucyl-tRNA synthetase (IleRS) and mupirocin. Each trace is the average of at least 2 repeats, with the transparent ribbon showing the standard deviation. The gray highlighted region demonstrates the terminal 30% time period that was used for averaging the final results, and the average and standard deviation for each repeat are shown as points with error on the right-hand side of each plot.

**Fig. 5.**
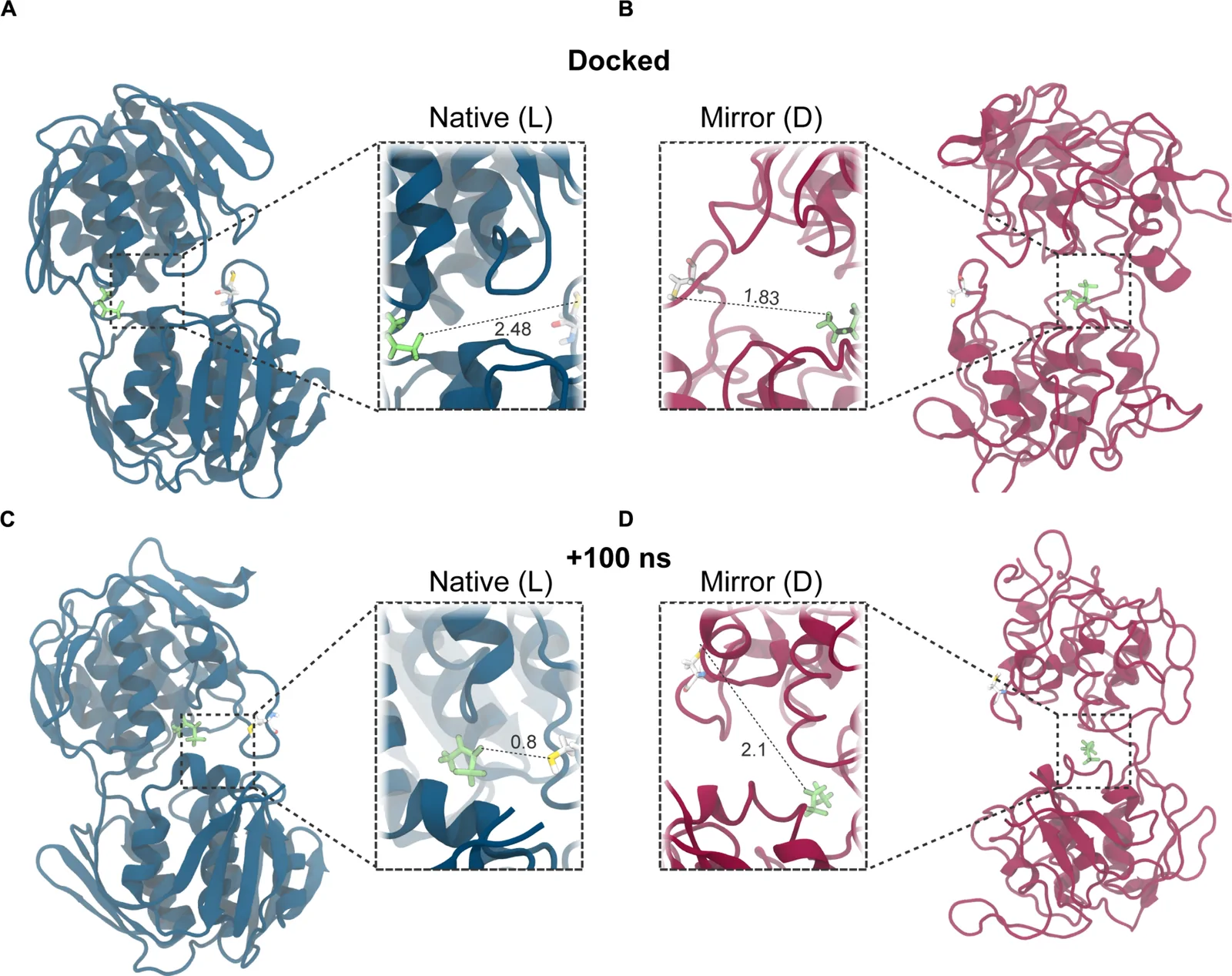
Mirror enolpyruvyl transferase (MurA) retains fosfomycin but cannot maintain a stable structure. (A) Equilibrated native MurA and (B) mirror MurA (e.g., D-form) docked with fosfomycin, with Cys115 highlighted. (C) Native and (D) mirror MurA after 100 ns of unrestrained dynamics with fosfomycin. Proteins are shown in the cartoon representation; native proteins are navy, and mirror forms are scarlet. Both antibiotics and key binding residues are depicted as sticks; all of the antibiotic molecules are shown in lime green, while the key binding residue is shown with carbon atoms in white, oxygen in red, nitrogen in blue, sulfur in yellow, and phosphorus in brown. Each zoomed inset shows the current distance between the closest part of the antibiotic and the side chain of the key binding residue (nm).

Once the antibiotic-bound forms of MurA were freely simulated, both native and mirror forms were able to retain the antibiotic. While FFQ was retained in the binding site of the mirror form MurA (Video S2), the antibiotic rapidly moved away from the target Cys115 residue (Fig. 4A (ii)), with an average distance of 2.06 ± 0.27, compared to 1.11 ± 0.06 nm in the native form (Fig. 3D). In nature, FFQ binding is dependent on the initial binding of UDP-GlcNAc to MurA, causing a conformational shift to enable PEP (or its antibiotic analog FFQ) to bind [52]. In this investigation, this did not appear to be necessary given the tight binding of the native protein to the antibiotic that we observed. Unlike the tight binding of the native form, the distance between the antibiotic FFQ and its target Cys115 was large enough that the covalent-bond-mediated inhibition by FFQ should not be able to occur, suggesting that the antibiotic may not be effective against mirror form MurA. However, in some simulations, the action of FFQ caused a marked destabilization of MurA (Fig. 5 and Video S2). This destabilization is demonstrated by the average ligand-bound RMSD: 0.15 ± 0.01 for the native *vs* 0.45 ± 0.17 nm for the mirror. This ligand-induced destabilization may also inhibit the enzyme’s function—but stands in stark contrast to the native action of the antibiotic, which induces a closed and nonfunctional state of the enzyme.

### Dihydropteroate synthase

DHPS is an enzyme involved in folate biosynthesis, essential in DNA synthesis and repair [53]. Folate synthesis is a pathway absent in humans, making DHPS a tractable antibacterial target exploited by a range of existing antibiotics. Here, we investigated DHPS from *S. aureus* [54], a human pathogen involved in a range of diseases including pneumonia, sepsis, and osteomyelitis, with a well-characterized and extensive antibiotic resistance profile. DHPS is a dimeric protein with 2 active sites, with flexible loop regions that contain antibiotic target residues Thr51, Phe17, and Ser18, which act to catalyze the condensation of 6-hydroxymethyl-7,8-dihydropterin-pyrophosphate and *p*-aminobenzoic acid to produce folate [55].

Equilibration simulations demonstrated rapid plateauing of both native and mirror form DHPS, suggesting geometric stability. The average RMSD values for DHPS in the absence of antibiotics were 0.21 ± 0.01 nm for the native form and 0.29 ± 0.001 nm for the mirror form (Fig. S1c). RoG was also used to confirm equilibration, with both forms demonstrating stability with average values of 2.68 ± 0.01 and 2.65 ± 0.008 nm (Fig. S1d). The RMSD values we obtained are similar, if slightly higher than previously reported [56], likely due to the use of different force fields and omission of stabilizing magnesium ions.

DHPS is inhibited by the antibiotic SMX. SMX is a competitive inhibitor that prevents *p*-aminobenzoic acid from accessing the binding site [57]. This inhibition is reversible, acting to sterically inhibit the natural substrate as opposed to inactivating key residues. However, mutation of key residues such as T51M, which is present in all pathogenic *S. aureus* DHPS variants [57], can inhibit SMX binding while still retaining enzyme function, resulting in clinical resistance [57]. This interaction between T51 and SMX guided the decision to measure the SMX–T51 distance as a proxy for antibiotic efficacy.

Docking of SMX into the native and mirror forms of DHPS showed very consistent results. The average docking scores were −5.67 ± 0.12 and −5.81 ± 0.18 kcal/mol (Fig. 3B) for the native and mirror forms, respectively. In both cases, the antibiotic was able to easily enter the active site of the protein. These values are in-line with previous computational work studying the binding of DHPS to other inhibitors, or myoglobin to an SMX analog, which yielded values between −6.04 and −7.42 kcal/mol, respectively [26,58].

Once bound to SMX, both native and mirror forms showed increased stability over the unbound forms (Fig. 3C). The average RMSD values for the native and mirror forms were 0.19 ± 0.003 and 0.28 ± 0.005 nm, respectively, suggesting stabilization of both forms, although the native state was marginally more stable. In both cases, the antibiotic is well retained in the active site, while it is not necessarily always close to the key residue selected (Thr51); this is not necessary for effective inhibition. The average distances between Thr51 and SMX were 1.4 ± 0.02 and 1.95 ± 0.05 nm for the native and mirror forms, respectively. In the native state, some reorganization of the antibiotic is visible (Video S3), which is not visible in the mirror form, allowing the antibiotic to be closer to T51. However, it is likely that in both cases, the activity of the enzyme would be sterically impeded.

### Penicillin-binding protein 3

PBP3 is an essential enzyme involved in peptidoglycan cross-linking during cell division [59]. The PBP3 form tested was from *C. trachomatis*, an obligate intracellular pathogen commonly involved in urogenital infections, which can result in chronic inflammation and scarring. PBP3 is a 73.67-kDa enzyme; however, only the 36.5-kDa TP domain was simulated [16]. The TP domain was selected both to reduce compute requirements and to confirm the simulation methodology used here gave results consistent with that of previous work from Pedroni *et al.*; i.e., mirror form antibiotic targets were geometrically stable but could not retain their antibiotic targets [11].

As with the previous proteins, the small TP domain rapidly reached geometric stability. The average RMSD values for PBP3 in the absence of antibiotics were 0.16 ± 0.02 nm for the native form and 0.38 ± 0.01 nm for the mirror form (Fig. S1e), consistent with the work of Pedroni *et al.* Further demonstration of equilibration came from the stable RoG of the native form (1.98 ± 0.01 nm), which was consistent with that of the mirror form (1.97 ± 0.02 nm), which had fully stabilized after the 100-ns equilibration, despite briefly spiking at 50 ns (Fig. S1f).

PBP3 was assessed with 2 different antibiotics, the widely used β-lactams AMX and CPX. Both antibiotics inhibit cell maturation and differentiation [60]. The antibiotics form a covalent adduct with Ser320 in the PBP3 active site, inhibiting peptidoglycan cross-linking [61]. The active site architecture, including the catalytic serine, of PBP is conserved in all PBP3 family proteins [62] although the residue numbering can differ [63]. This high degree of conservation in PBP3 proteins, which are essential in gram-negative bacterial cell division, makes it an attractive target for broad-spectrum β-lactam antibiotics.

Docking PBP3 in complex with AMX and CPX showed comparable binding strengths. In all cases, the antibiotic could fit into the active site of both the native and mirror forms. The average docking scores of the top 3 docking poses were −6.88 ± 0.23 and −6.31 ± 0.54 kcal/mol for the native and mirror PBP3 binding to AMX and −5.01 ± 0.44 and −4.96 ± 0.24 kcal/mol for CPX (Fig. 3B). Experimental data for AMX and CPX binding to PBP3 reveal IC_50_ values of 3 and 30 μg/ml, respectively, which would translate to binding affinities of −7.3 and −5.8 kcal/mol at 25 °C, in-line with the simulated values [64].

**Fig. 6.**
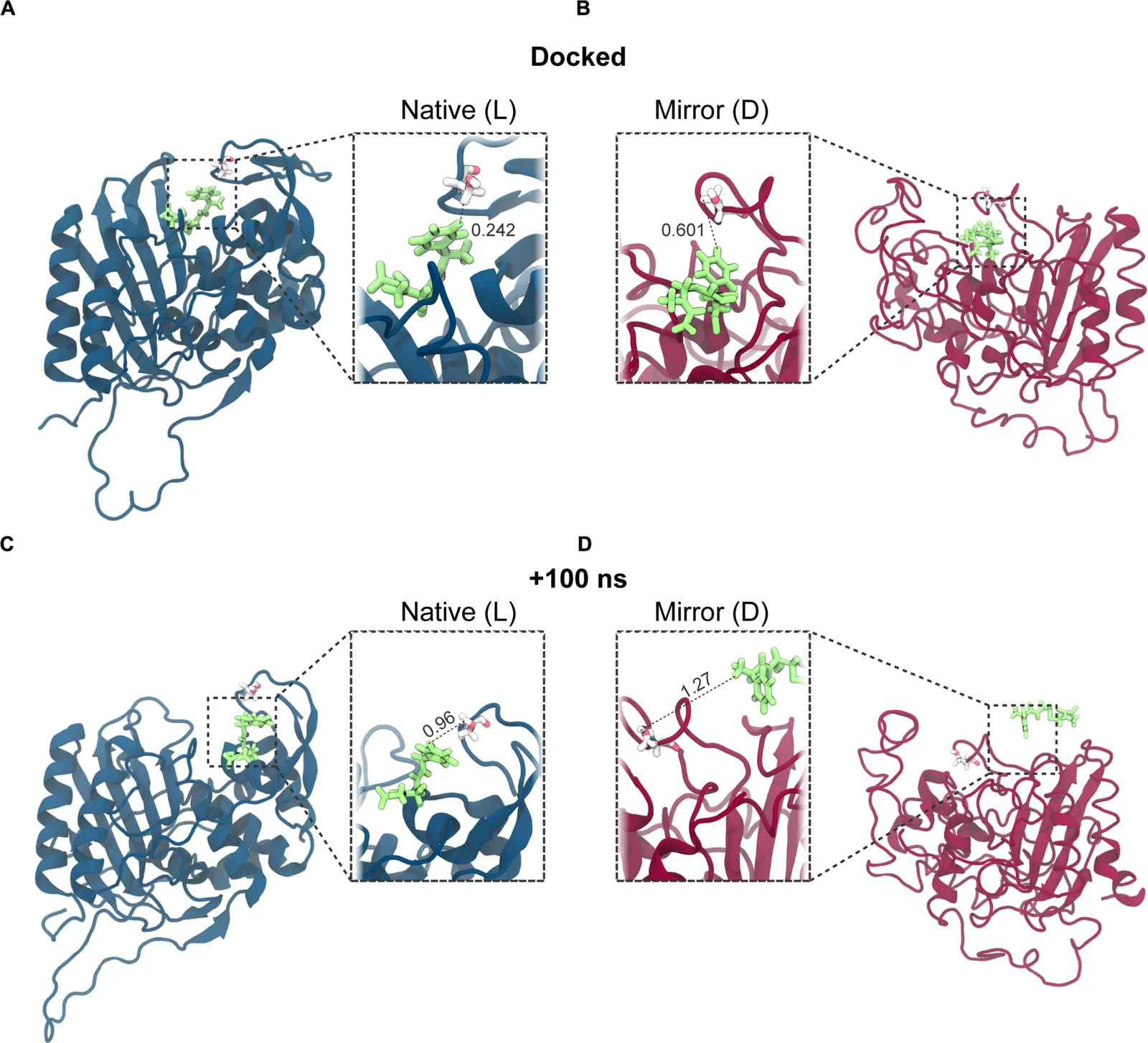
Mirror penicillin-binding protein 3 (PBP3) cannot retain cephalexin within the binding pocket. (A) Equilibrated native PBP3 and (B) mirror PBP3 (e.g., D-form) docked with cephalexin, with Ser320 highlighted. (C) Native and (D) mirror PBP3 after 100 ns of unrestrained dynamics with cephalexin. Proteins are shown in the cartoon representation; native proteins are navy, and mirror forms are scarlet. Both antibiotics and key binding residues are depicted as sticks; all of the antibiotic molecules are shown in lime green, while the key binding residue is shown with carbon atoms in white, oxygen in red, nitrogen in blue, sulfur in yellow, and phosphorus in brown. Each zoomed inset shows the current distance between the closest part of the antibiotic and the side chain of the key binding residue (nm).

When simulated with the docked antibiotics, both the native and mirror proteins were stable, but the mirror form was unable to retain the antibiotics in close proximity. Protein stability was consistent across repeat simulations with RMSD values of 0.27 ± 0.02 and 0.31 ± 0.02 nm for the native and mirror forms complexed to AMX, respectively, and 0.26 ± 0.004 and 0.38 ± 0.02 nm for the native and mirror forms complexed to CPX, respectively (Fig. 3C). Despite this stability, the distance between the antibiotics and their target residue Ser320 demonstrated the ineffectiveness of the antibiotics against the mirror form bacterial target. In the native forms, AMX was retained within 0.99 ± 0.3 nm of Ser320, while CPX was 1.31 ± 0.52 nm (Fig. 4C (ii) and D (ii)). However, the mirror forms saw the antibiotics exit the binding site within 10 ns for CPX and 60 ns for AMX, with final distances being 5.54 ± 0.3 nm for AMX and 4.67 ± 1.4 nm for CPX (Fig. 4C (ii) and D (ii)). This strongly suggests that the antibiotics would be ineffective. Although it may be feasible that AMX could have some long-lasting inhibitory action in the 60 ns that it was retained in the binding site, it is evidently not well retained compared to the native form of the protein (Fig. 6).

The computational process used to generate the mirror proteins could also be performed on the antibiotics, so a chiral mirrored form of AMX was assessed against native and mirror PBP3. When tested, our results were comparable with previous work [12], where the mirrored antibiotic could be retained in the mirrored protein. Building on previous work, we also investigated the efficacy of the mirror antibiotic on native PBP3, in which predictably the mirror antibiotic was not well retained in the active site of the native protein (Fig. S5). The mirrored chirality antibiotic was retained within active site of the mirror PBP3, 1.7 ± 1.9 nm from Ser320, compared to the 2.1 ± 1.3 nm in the native form.

### Isoleucyl-tRNA synthetase

IleRS is an essential aminoacyl tRNA synthase enzyme. It acts to assemble isoleucine–tRNA complexes involved in protein synthesis by transferring aminoacyl adenylate to the 3′ terminal adenosine of tRNA [65]. The IleRS investigated here is from the thermophilic eubacterium *T. thermophilus*, selected for the high-quality crystallography data in complex with the antibiotic MRC [17]. While *T. thermophilus* is not a human pathogen, tRNA synthesis is highly conserved and there is no substantive difference in the amino acid sequence between the catalytic domains of IleRS proteins from different kingdoms [65].

As the largest protein studied, IleRS showed some of the highest RMSD and RoG values but still appeared to rapidly reach geometric equilibrium. The 95.31-kDa protein appeared to reach stability in both native and mirror forms within the first 10 ns, and RMSD and RoG were consistent from that time onward. The average RMSD for the native IleRS was 0.29 ± 0.03 nm, and the mirror form again showed a slightly higher RMSD at 0.35 ± 0.001 nm (Fig. S1g). Despite the small change in stability induced by computational mirroring, the RoG values were consistent with both native and mirror forms showing 3.46 ± 0.002 and 3.46 ± 0.003 nm for the native and mirror, respectively (Fig. S1h).

IleRS is inhibited by the action of the antibiotic MRC. MRC is an analog of the isoleucyl-adenylate substrate, competitively excluding it from the active site and disabling protein synthesis [65]. MRC is believed to bind to Leu583 in the active site, as its substitution in eukaryotic IleRS confers resistance to MRC [65,66]. This lack of efficacy against eukaryotic IleRS and the relatively high degree of conservation of Leu583 in bacterial homologs [67] make MRC an effective antibiotic.

The docking of MRC into the active site of IleRS showed the largest difference between the native and mirror states of any of the proteins tested. Native IleRS showed highly consistent docking, with a binding energy of −6.5 ± 0.11 kcal/mol. However, the mirrored form was highly inconsistent; the top docking pose had a comparable binding energy to the native (Fig. 3B), but the rest were bound substantially less strongly, resulting in an average binding energy of −2.2 ± 3 kcal/mol. Despite the binding energy being substantially reduced compared to that of the native protein, it was still spontaneous, suggesting that binding would occur. The pose with a docking score of −6.5 kcal/mol was carried forward for further simulation as it is comparable with previously published data for the IleRS–MRC binding affinity: −7.6 kcal/mol [68].

IleRS from *T. thermophilus* is the only protein tested to show comparable binding and stability to its cognate antibiotic between the native and mirrored forms. The antibiotic bound trajectories had comparable RMSDs (Fig. 3C) of 0.29 ± 0.08 and 0.31 ± 0.03 nm for the native and mirror states, respectively, nearly identical to the unbound RMSD. The average distances between MRC and its target residue, Leu583, were 1.28 ± 0.19 and 1.71 ± 0.1 nm in the native and mirror states, respectively (Fig. 3D), with the antibiotic staying tightly bound into the active site in both the mirror and native forms (Figs. 4E (ii) and 7). This may be due to the highly flexible nature of both MRC and IleRS. IleRS is highly flexible, a known feature of proteins from thermophilic species (Fig. 2B) [69]. However, the variable docking scores suggest that it may be unlikely for the antibiotic to spontaneously enter the binding site, even if it can be retained once there. It is interesting that the only instance of possible antibiotic effectiveness against both native and mirror forms in our work comes from a species that is not pathogenic to humans. It is possible this effect derives from the extremophilic nature of the protein. Extremophilic proteins tend to show increased local flexibility (Fig. 2D) [70], rendering ligand-binding pockets more tolerant to structural change [71,72]. In conjunction, the long-carbon chain of MRC (Fig. 2B) also confers greater flexibility, which may also enhance binding tolerance [73].

**Fig. 7.**
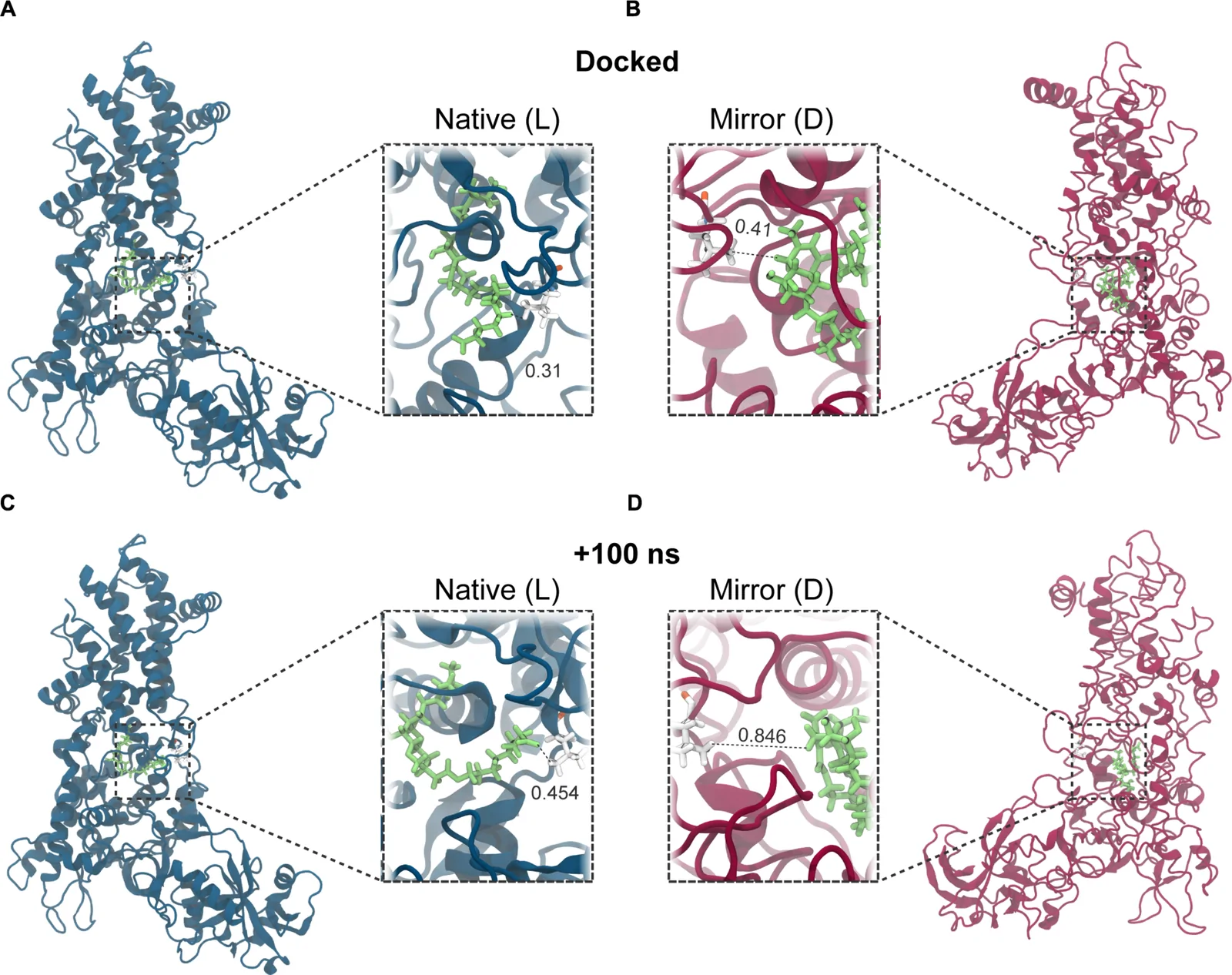
Flexible thermophilic pathogen targets were better able to retain their antibiotic ligand. (A) Equilibrated native isoleucyl-tRNA synthetase (IleRS) and (B) mirror IleRS (e.g., D-form) docked with mupirocin, with Leu583 highlighted. (C) Native and (D) mirror IleRS after 100 ns of unrestrained dynamics with mupirocin. Proteins are shown in the cartoon representation; native proteins are navy, and mirror forms are scarlet. Both antibiotics and key binding residues are depicted as sticks; all of the antibiotic molecules are shown in lime green, while the key binding residue is shown with carbon atoms in white, oxygen in red, nitrogen in blue, sulfur in yellow, and phosphorus in brown. Each zoomed inset shows the current distance between the closest part of the antibiotic and the side chain of the key binding residue (nm).

## Discussion

In most cases, antibiotics were not able to remain in close proximity to the binding pockets of the mirror forms of bacterial proteins—unlike native forms, despite initially favorable docking. Docking results showed spontaneous interaction for all cases and, in all examples apart from mirrored IleRS, were highly consistent between native and mirror proteins. The docking scores were further supported by molecular mechanics/Poisson–Boltzmann surface area (MM/PBSA) analysis of the ligand-bound trajectories, where the free energy calculations agreed with the docking scores, further suggesting spontaneous binding (Fig. S7). It should be noted that the trajectories generated here sampled only the lowest-energy docking poses, and further sampling may reveal more of the energetic landscape. However, the high consistency of docking scores, and the substantial conformational relaxation time for both the ligand and receptor, reduces the dependence of the conclusions on the initial docking geometry. In many cases (Fig. 4B (ii)), substantial spatial rearrangement occurred until a stable ligand–receptor equilibrium was reached.

From these data, it may be inferred that the antibiotics tested would fail to exert an antimicrobial effect on potential future mirror bacteria. However, it must be noted that these simulations were performed using a nonreactive force field, CHARMM36, which does not explicitly model bond-breaking or bond-formation events. As a result, these data may understate the effectiveness of antibiotics that rely on covalent modification of their targets, such as AMX. More rigorous approaches using QM/MM simulations of reactive force fields (e.g., ReaxFF) exist but are associated with increased compute cost and parameterization challenges, particularly for the large biomolecular systems studied here. As such, classical force fields are still widely used for studying ligand–protein binding kinetics [74] and have been well validated experimentally [75]. Thus, this work provides insight into whether the antibiotic ligands and their target proteins can adopt the stable binding configurations required for subsequent covalent modification steps.

This study has a number of limitations, in particular that it was a small-scale computational study. Longer simulations, of a wider range of antibiotics and protein targets, with more exhaustive conformational sampling or ensemble-based approaches would bolster the findings here. While these were beyond the scope of this exploratory study, we hope that our findings motivate further research by other groups. These simulations evaluate isolated protein–ligand interactions only; whole-cell susceptibility additionally depends on membrane permeability, transporter uptake, efflux, metabolism, and cell-envelope architecture, which may differ substantially in mirror bacteria and are not captured here; for instance, SMX efficacy also depends on pathway-level folate metabolism beyond DHPS binding. This limits our ability to infer or draw strong conclusions regarding the ultimate antimicrobial efficacy of existing countermeasures against mirror bacteria. However, our work sits within a small but growing corpus of literature providing preliminary data on interactions between mirror bacterial macromolecules and existing countermeasures [11,12] and proxy data on mirror countermeasures and existing bacteria [10]. While imperfect models, these studies have broadly demonstrated lower binding affinities and efficacies.

Ideally, future work would also include experimental data to validate the relevance of the computational methodology we have adapted and employed. Both the present study and previous work from Pedroni *et al.* form computational mirror systems via coordinate inversion. This forms geometrically valid mirror counterparts, and our data have indicated that it well preserves the structural organization of the proteins (Fig. S2); however, as this work is purely computational, it cannot establish that this corresponds to experimentally producible proteins. The CHARMM36 force field has been extensively parameterized and optimized for L-amino acid form proteins, and while bonded interactions are formally invariant, stereochemical inversion here appears to have slightly altered dihedral bond energies (Fig. S3)—although within measurement error, likely affecting subsequent side-chain packing geometries, which could have knock-on effects on the hydrogen-bonding networks and solvent organization of the proteins. This work does not suggest that these changes would arise under *de novo* folding of mirror proteins and instead focuses on comparative analysis of the mirrored ligand–target interactions within a consistent computational framework. Further work that validates the realism and behavior of mirror proteins would further establish this precedent, as well as enable the optimization of computational force fields for mirror proteins—but may also act as an enabler in the development of physical mirror life-forms and the potential threats that they represent. There are already early attempts to define the efficacy of existing medical countermeasures against mirror bacteria [10]. Further work of this kind, in addition to *in vitro* binding kinetics experiments between mirror proteins and antibiotics, would help to confirm our findings.

While most antibiotics were shown to be ineffective, antibiotics that act through steric hindrance (as opposed to covalent modification) appear to have greater potential against mirror life, as the conditions for effectiveness are often less spatially stringent. This is likely to depend on the size and flexibility of the binding pocket. This is worrying as antibiotics that work by covalent modification are often more potent and selective antibiotics [76], as well as one of the few types of antibiotics in which we continue to see discoveries [77]. Some of the most historically important and widely used antibiotics, such as β-lactams, work through covalent modification of target proteins [78]. Antibiotics that are highly flexible, or target flexible proteins, are likely to be more effective against mirror life. This is a positive effect that warrants further investigation as part of any future antimirror drug development efforts. These could draw on existing drug development strategies, as many antibiotics such as linezolid and related oxazolidinones have been optimized by increasing their rotational freedom [79]. Further, many more modern antibiotics (especially peptide mimics) [80] are highly flexible and may as a result be more effective against mirror life.

Despite the limitations of exploratory computational data presented here, our findings add to the list of experiments that suggest that existing antibiotics may not be effective against mirror bacteria. The creation of mirror life represents a serious biosecurity threat; the extent to which we would be able to mitigate this threat with existing medical countermeasures warrants substantial further investigation.

## Data Availability

All trajectories are available upon request.

## Supplementary Materials

Supplementary 1 Figs. S1 to S7 Tables S1 to S4 Videos S1 to S10 Reference [90]
