# Supplementary material for: Molecular Dynamics Simulations Demonstrate Reduced Antibiotic Affinity to Mirror Bacterial Targets
Source: Comput Struct Biotechnol J. 2026 Sep 3;35(1):0198. doi: 10.34133/csbj.0198 (PMC13538942; doi:10.34133/csbj.0198)
Supplement: Supplementary file 1 [file csbj.0198.f1.zip › Supplementary Materials.docx]

**Supplementary Materials**

**Figure S1.** **Native and mirror form proteins reached geometric equilibrium after 100 ns. (a)** Time-averaged alpha carbon RMSD and **(b)** RoG for MurA, **(c-d)** DHPS, **(e-f)** PBP3 and (**g-h)** IleRS. Native proteins are shown in navy and mirror in scarlet. Each trace is an average of 2 repeats and the coloured ribbon shows the standard deviation. **(i)** Average RMSD and **(j)** RoG from the final 30% of each trajectory. Each graph shows the average of at least two repeats, the error bars show the standard deviation and the points show the raw data.

**Figure S2. Computational mirroring of proteins retains most secondary structure.** Using DSSP (Dictionary of Secondary Structure in Proteins),^[90](https://www.zotero.org/google-docs/?yMCzl8)^ the percentage of each protein that exists as a loop, alpha helix or beta strand was calculated for the equilibration simulations. Each trace is an average of two repeats, and the coloured ribbon shows the standard deviation.

**Figure S3. Improper Dihedral Energy for representative protein and mirror counterpart show very little difference.** Each line is an average of 2 independent repeats, and the coloured ribbon shows the standard deviation, a 1 ns running average has been applied.

**SI Figure S4. A representative example demonstrating 100 ps is adequate for (a) isothermal (b) isobaric equilibration.**

**Figure S5. Stability and Ser320 distance for PBP3 when binding to mirrored amoxicillin. (a)** Alpha carbon RMSD of native PBP3 (blues) and the mirror form (reds) after docking with a mirrored amoxicillin. **(b)** Distance between the centre of mass of amoxicillin and Ser320, a key binding residue in PBP. Each individual trace is shown, with a 2 ns running average, with the coloured ribbon showing the standard deviation. To determine equilibrium values for comparison, the final 30% (shown in grey) of each trajectory was averaged to reveal; RMSD values of 0.34 ± 0.06 and 0.23 ± 0.04 nm, and a centre of mass distance between mirrored amoxicillin and Ser320 of 2.05 ± 1.25 and 1.72 ± 1.9 nm for the native and mirror forms respectively, shown as points to the right of the plots. **(c)** shows the structural formula of the computationally mirrored amoxicillin.

**Figure S6. Demonstration of computational chiral inversion. (a)** Structural formula of an isoleucine amino acid showing the L (native) and D (mirror) forms on the left and right respectively. The chiral centres are indicated with a golden star. **(b)** schematic render of an isoleucine residue that has been through the computational chiral inversion, where the x, y, z coordinates of each atom are converted to -x, -y, -z. Carbon atoms are coloured grey, hydrogen white, nitrogen blue and oxygen red. Note that the structure in b is missing the monomer hydrogens shown in a as it is part of an amino acid polymer.

**SI Figure S7. MM/PBSA analysis of docked trajectories agrees with docking scores.** Binding free energies were estimated using the MM/PBSA approach implemented in g_mmpbsa and was performed on a 20 ns snapshot at the end of the ligand-bound trajectory of the native form proteins, sampled at 1 ns intervals. A protein dielectric constant of 4 was used throughout, polar solvation energies were obtained by numerical solution of the Poisson–Boltzmann equation, while non-polar contributions were calculated from solvent-accessible surface area terms. Average scores are shown as bars, with individual data as points and the standard deviation shown as error bars.

# Table S1 Root Mean Squared Deviation

| **Protein** | **Ligand** | **Enantiomer** | **RMSD (nm)** | **StDev** |
| --- | --- | --- | --- | --- |
| MurA | Unbound | Native | 0.16 | 0.02 |
| MurA | Unbound | Mirror | 0.35 | 0.1 |
| DHPS | Unbound | Native | 0.21 | 0.009 |
| DHPS | Unbound | Mirror | 0.29 | 0.001 |
| PBP3 | Unbound | Native | 0.16 | 0.016 |
| PBP3 | Unbound | Mirror | 0.38 | 0.012 |
| IleRS | Unbound | Native | 0.29 | 0.028 |
| IleRS | Unbound | Mirror | 0.35 | 0.001 |
| MurA | Fosfomycin | Native | 0.15 | 0.012 |
| MurA | Fosfomycin | Mirror | 0.45 | 0.167 |
| DHPS | Sulfamethoxazole | Native | 0.19 | 0.003 |
| DHPS | Sulfamethoxazole | Mirror | 0.28 | 0.005 |
| PBP3 | Amoxicillin | Native | 0.27 | 0.02 |
| PBP3 | Amoxicillin | Mirror | 0.31 | 0.022 |
| PBP3 | Cephalexin | Native | 0.26 | 0.004 |
| PBP3 | Cephalexin | Mirror | 0.38 | 0.024 |
| IleRS | Mupirocin | Native | 0.29 | 0.08 |
| IleRS | Mupirocin | Mirror | 0.31 | 0.034 |
| PBP3 | Mirror-Amoxicillin | Native | 0.34 | 0.06 |
| PBP3 | Mirror-Amoxicillin | Mirror | 0.23 | 0.04 |

**Table S2 Radius of Gyration**

| **Protein** | **Enantiomer** | **RoG (nm)** | **StDev** |
| --- | --- | --- | --- |
| MurA | Native | 2.23 | 0.004 |
| MurA | Mirror | 2.31 | 0.019 |
| DHPS | Native | 2.68 | 0.012 |
| DHPS | Mirror | 2.65 | 0.008 |
| PBP3 | Native | 1.98 | 0.012 |
| PBP3 | Mirror | 1.97 | 0.015 |
| IleRS | Native | 3.46 | 0.002 |
| IleRS | Mirror | 3.46 | 0.003 |

**Table S3 Docking Scores**

| **Protein** | **Ligand** | **Enantiomer** | **DockingScore (kcalmol-1)** | **StDev** |
| --- | --- | --- | --- | --- |
| PBP3 | Amoxicillin | Native | -6.88 | 0.23 |
| PBP3 | Amoxicillin | Mirror | -6.31 | 0.54 |
| PBP3 | Cephalexin | Native | -5.01 | 0.44 |
| PBP3 | Cephalexin | Mirror | -4.96 | 0.24 |
| IleRS | Mupirocin | Native | -6.5 | 0.11 |
| IleRS | Mupirocin | Mirror | -2.15 | 3.00 |
| DHPS | Sulfamethoxazole | Native | -5.67 | 0.12 |
| DHPS | Sulfamethoxazole | Mirror | -5.81 | 0.18 |
| MurA | Fosfomycin | Native | -4.13 | 0.24 |
| MurA | Fosfomycin | Mirror | -4.12 | 0.28 |

**Table S4 Key Residue Distance**

| **Protein** | **Ligand** | **Enantiomer** | **KeyResidue** | **Distance (nm)** | **StDev** |
| --- | --- | --- | --- | --- | --- |
| MurA | Fosfomycin | Native | CYS_115 | 1.11 | 0.062 |
| MurA | Fosfomycin | Mirror | CYS_115 | 2.06 | 0.27 |
| DHPS | Sulfamethoxazole | Native | THR_51 | 1.4 | 0.021 |
| DHPS | Sulfamethoxazole | Mirror | THR_51 | 1.95 | 0.05 |
| PBP3 | Amoxicillin | Native | SER_320 | 0.99 | 0.3 |
| PBP3 | Amoxicillin | Mirror | SER_320 | 5.54 | 0.28 |
| PBP3 | Cephalexin | Native | SER_320 | 1.31 | 0.53 |
| PBP3 | Cephalexin | Mirror | SER_320 | 4.67 | 1.38 |
| IleRS | Mupirocin | Native | LEU_583 | 1.28 | 0.19 |
| IleRS | Mupirocin | Mirror | LEU_583 | 1.71 | 0.012 |
| PBP3 | Mirror-Amoxicillin | Native | SER_320 | 2.05 | 1.25 |
| PBP3 | Mirror-Amoxicillin | Mirror | SER_320 | 1.72 | 1.9 |
